# Clinical Lessons on Nervous Diseases

**Published:** 1897-08

**Authors:** 


Clinical Lessons on Nervous Diseases. By S. Weir Mitchell, M. D., LL. D., Edin., Member of the National Academy of Sciences; Honorary Fellow of the Royal Medico-Chirurgical Society of London. Handsome 12mo, 299 pages, with illustrations and two colored plates. Cloth, $2.50. Philadelphia and New York: Lea Brothers & Co., Publishers. 1897.

The present volume is the work of a careful, painstaking observer, who finds two great reasons for bringing forth a book on this subject. First, the great importance of the subject; and, second, that although there exists much literature on the subject, scattered through the medical press, yet there is no recent book in English devoted to its consideration. The object of the work is to give the general practitioner a careful, concise statement of the symptomatology, the differential diagnosis and the various therapeutic measures that have been found useful in the treatment of these disorders. It will not be amiss to state at the outset that the author has succeeded admirably in his undertaking.

The historical consideration of hysteria has always been a most interesting one and the several epidemics of hysteria, breaking out among the numerous races, at various intervals, is instructive reading.

Under the head of symptomatology, the author takes up the (1) disturbances of sensation; (2) disturbances of motion; (3) vasomotor, visceral and nutritive disturbances; (4) mental symptoms, and (5) the large number of miscellaneous symptoms which do not come under either of the foregoing classes.

The treatment is what must interest the general practitioner, and if he follows the instructions given by the author he cannot fail to get good results.

The work is admirably gotten up, the illustrations are excellent and, on the whole, is a valuable addition to neurological literature.

